# Functional Analysis of Rhythmic Jaw Movements Evoked by Electrical Stimulation of the Cortical Masticatory Area During Low Occlusal Loading in Growing Rats

**DOI:** 10.3389/fphys.2020.00034

**Published:** 2020-01-31

**Authors:** Phyo Thura Aung, Chiho Kato, Yasunori Abe, Takuya Ogawa, Hideyuki Ishidori, Akiyo Fujita, Hidemasa Okihara, Satoshi Kokai, Takashi Ono

**Affiliations:** Department of Orthodontic Science, Graduate School of Medical and Dental Sciences, Tokyo Medical and Dental University, Tokyo, Japan

**Keywords:** rhythmic jaw movements, cortical masticatory area, intracortical microstimulation, electromyography, soft diet, rat

## Abstract

The maturation of rhythmic jaw movements (RJMs) and related neuromuscular control has rarely been studied in animals, though this process is essential for regulating the development of stomatognathic functions. Previous studies have shown that occlusal hypofunction during growth alters masticatory performance. However, little is known about patterns of cortically-induced RJMs under conditions of soft-diet feeding during development. The aim of this study is to clarify the effect of low occlusal loading on the pattern of cortically induced RJMs and related neuromuscular responses in growing rats. Sixty-four 2-week-old male albino Wistar rats were randomly divided into two groups and fed on either a normal diet (control) or soft diet (experimental) soon after weaning. At 5, 7, 9, and 11 weeks of age, electromyographic (EMG) activity was recorded from the right masseter and anterior digastric muscles along with corresponding kinematic images in RJMs during repetitive intracortical microstimulation of the left cortical masticatory area (CMA). Rats in both groups showed an increase in gape size and lateral excursion until 9 weeks of age. The vertical jaw movement speed in both groups showed no significant difference between 5 and 7 weeks of age but increased with age from 9 to 11 weeks. Compared to the control group, the average gape size and vertical speed were significantly lower in the experimental group, and the pattern and rhythm of the jaw movement cycle were similar between both groups at each recording age. EMG recordings showed no age-related significant differences in onset latency, duration, and peak-to-peak amplitude. Moreover, we found significantly longer onset latency, smaller peak-to-peak amplitude, and greater drop-off mean and median frequencies in the experimental group than in the control group, while there was no significant difference in the duration between groups. These findings indicate that a lack of enough occlusal function in infancy impedes the development of patterns of RJMs and delays the neuromuscular response from specific stimulation of the CMA.

## Introduction

Mastication involves complex neuromuscular interactions between central and peripheral control mechanisms. The masticatory process is assisted by the coordinated movement of the tongue, jaw, and masticatory muscles, followed by the appropriate positioning of the jaw during opening and closing (Nakamura and Katakura, 1995). With growth-related changes, modifications of masticatory functions occur in response to varying functional demands. The critical step for the development of mastication appears to begin around the weaning period because the main feeding process changes from sucking to chewing. Subsequently, many morphological changes occur, including tooth eruption, an increase in muscle mass and jaw growth, and maturation of the nervous system (Herring, 1985).

Masticatory movements are generated by neural mechanisms originating in the brain. Research into typical rhythmic jaw movements (RJMs) is essential for understanding the development of masticatory functions in mammals. RJMs are generated by interconnected neural systems termed the masticatory central pattern generator (CPG), which is located in the brainstem (Lund, 1991). In order to produce the complex and variable patterns of mastication-like behavior, the CPG can be modulated by inputs from peripheral afferents as well as higher centers such as the cortical masticatory area (CMA) (Lund et al., 1981; Lund, 1991; Nakamura and Katakura, 1995). RJMs can be generated at the cortical level through repetitive intracortical microstimulation of the CMA (Avivi-Arber et al., 2011; Avivi-Arber and Sessle, 2018). In addition, intracortical microstimulation of the CMA can evoke orofacial movements and electromyographic (EMG) activity within the orofacial muscles (Neafsey et al., 1986). The CMA is composed of areas encompassing the sensorimotor cortex, which also evokes RJMs when it is stimulated with repetitive electrical stimulation. Several studies have reported that different areas of the CMA evoke different RJM patterns and jaw muscle EMG activity in rats (Sasamoto et al., 1990; Satoh et al., 2007; Maeda et al., 2014), guinea pigs (Isogai et al., 2012), rabbits (Lund et al., 1984), and monkeys (Huang et al., 1989). Moreover, stimulation of the postero-lateral region of the CMA induces grinding movements that resemble natural chewing patterns in rats (Sasamoto et al., 1990).

Daily masticatory muscle activity is associated with the development of the masticatory system. Currently, modern dietary forms tend to be soft and easily digested, which dramatically influences the growth of a normal stomatognathic system (Proffit et al., 1998). Prolonged changes in masticatory function could lead to altered craniofacial morphology (Hichijo et al., 2014; Kufley et al., 2017), development of muscle fibers of masticatory muscles (Grunheid et al., 2009; Kawai et al., 2010), and neuronal connections in the hippocampus (Okihara et al., 2014; Anegawa et al., 2015). Recent studies have suggested that changes in masticatory function have an influence in motor representations within the sensorimotor cortex (Avivi-Arber et al., 2010a, b, 2015). With regards to masticatory functions, previous studies have shown that changes in loading force produce alterations in masticatory patterns and rhythm when chewing normal or soft food (Yamada et al., 2006; Fujishita et al., 2015). However, the development of RJMs in association with the CMA have been rarely described to date, in relation to changing occlusal function during maturation.

Our aim was to evaluate the developmental course of specific RJMs that are evoked by stimulation of the CMA, and to examine the effect of changing occlusal loading on cortically-induced RJMs and associated neuromuscular responses during development. Jaw movement regulation is of key importance for masticatory performance during early chewing. Thus, we hypothesized that the effect of decreased occlusal loading through feeding with a soft diet might negatively affect the regulation of RJMs evoked by the CMA during development. We examined both jaw movement trajectories and EMG activities of the jaw muscles of rats fed on a soft diet. These findings were compared with a control group fed a normal diet and across age groups to examine whether low occlusal loading impeded the maturation of jaw movements during the period when the development of a mechanism of mastication is activated.

## Materials and Methods

### Animal Model and Surgical Preparation

This study was performed according to the recommendations of the guide for the Institutional Animal Care and Use Committee (A2017-135A and A2018-028A) in compliance with the Animal Care Standards of Tokyo Medical and Dental University.

Sixty-four albino Wistar rats were used in this study (male, 2-weeks-old). All young cubs were examined and confirmed to be weaning to prevent any experience of chewing a solid diet from being included in the experimental group. Immediately after weaning, infants were divided into two equally-sized groups receiving: normal diet (control), fed with ordinary chow pellet (CE-2, CLEA, Inc., Tokyo, Japan) (*n* = 32); and soft diet (experimental), fed with powder pellet (<0.02 mm diameter) (*n* = 32) until 11 weeks of age. Both groups were weighed weekly throughout the entire experimental period.

All rats underwent the experimental procedure at 5, 7, 9, or 11 weeks of age (*n* = 8 per group per time point). Ketamine-HCl [100 mg/kg, intraperitoneal (IP)] injection was administrated initially for the craniotomy and EMG electrode insertion. Supplementary doses were injected whenever necessary to maintain a constant level of anesthesia according to checks of vibrissa movements, pinch-withdrawal, and corneal reflexes throughout the experiments. The local anesthetic lidocaine hydrochloride (2%) was injected into the subcutaneous space below the planned surgical areas. Body temperature was maintained at 37–38°C using a thermo-regulated heating pad.

To record EMG activity from the jaw muscles, a midline incision was made along the neck from the mandible on the ventral surface to the rostral portion to expose the right side of masseter (jaw-closer) and anterior digastric (jaw-opener) muscles. Bipolar EMG electrodes (40 gauge, single-stranded, Teflon insulated stainless-steel wires, 2 mm inter-electrode distance) were then inserted into the muscles to record EMG activity.

To stimulate the cerebral cortex, part of the left frontal and parietal bones were drilled using a dental bar to expose the outer surface of the masticatory cortex, and the dura mater was covered with paraffin liquid oil (37°C). The lower incisors were ground to approximately 1 mm to minimize interference of jaw and tongue movements, especially at 9 and 11 weeks of age. The rat was then secured in a stereotaxic apparatus (models SN-2 and Sm-15M; Narishige Scientific Instruments, Tokyo, Japan). A fine glass-insulated tungsten microelectrode (shaft diameter 100 μm, impedance 1–3 MΩ at 1 kHz; Unique Medical, Tokyo, Japan) was inserted vertically into the masticatory area of the cerebral cortex. Electrical stimulation (0.5 ms duration, 20 Hz, 180 μA, 7 s) was applied to the left CMA (0–2 mm rostral, 4–5.5 mm lateral to Bregma, 4–5 mm ventral). A reference electrode was attached to the exposed neck muscle. RJMs were determined by the mandibular movements and rhythmic bursts of the anterior digastric and masseter EMG activities. Three trials of stimulation were performed at each stimulation site.

### Recording of Jaw Movements and EMG Activity

For recording sessions, a wire (0.7 mm thick) attached to a marker was placed between the lower incisors attached with dental resin. A digital high-speed HAS-U1M camera (DITECT, Corp., Tokyo, Japan) was set directly in front of the marker to detect jaw movements. During stimulation, a videotape of the jaw movements was recorded, and 2D motion analysis system software (Dipp-motion V, DITECT, Cop., Tokyo, Japan) was used to refine the marker position of the jaw movements.

Signals of EMG activity were filtered and amplified using a multichannel amplifier (MEG-6108; Nihon Kohden, Tokyo, Japan; 1000x gain, bandpass 0.3–3 kHz), and all EMG waveforms were rectified and averaged. Data were analyzed offline using the CED 1401 interface and Spike2 software for Windows, version 5.21 (Cambridge Electronic Design, Cambridge, United Kingdom). EMG responses of each muscle were analyzed in a fast sweep. To analyze the specific characteristics of the EMG, the sonogram was calculated based on the peak root-mean-square EMG activity, and the power spectrum was displayed using fast Fourier transform.

The RJMs and EMG activities were stored on a computer disk. Jaw movement patterns during stimulation were observed from the frontal plane. The jaw movement parameters measured were: (1) gape size (vertical excursion between maximum opening and maximum closing), (2) lateral excursion (horizontal distance between minimum jaw-opening position and the most lateral jaw position), (3) speed of vertical jaw movements (rate of jaw movement distance per s), (4) changes in the vertical jaw movement pattern (the 13 points traced with an interval of 10 ms along the path of one cycle of jaw movement), (5) jaw-opening duration (time between maximum closing and subsequent maximum opening), (6) jaw-closing duration (time between maximum opening and subsequent maximum closing), (7) total cycle duration (duration between two consecutive maximum openings), (8) rhythm of jaw movements during stimulation (total cycle duration of the jaw movement during stimulation at each time point per s), (9) changes in amplitude of vertical jaw movements (gape size) in relation to the time in the sequence (total duration of stimulation divided into three equal periods: early, middle, and late, corresponding to the first, second, and third, for 2 s respectively). Jaw muscle activity was analyzed in terms of (1) onset latency (interval between the onset of the stimulus and the onset of the first response), (2) duration (interval between the onset and offset of the first response), (3) peak-to-peak amplitude (amplitude from baseline to the positive peak of the first response), (4) median frequency from the power spectrum (the EMG power spectrum divided into two halves with equal amplitude) and (5) mean frequency of the power spectrum (average frequency which is calculated as the sum of product of the EMG power spectrum and the frequency divided by the total sum of the power spectrum) (Phinyomark et al., 2012). The mean values of data for each parameter were measured from 10 chewing cycles. The EMG bursts were identified when the rectified EMG exceeded the mean by 2SD. All values are expressed as mean ± standard deviation.

### Statistical Analysis

Significant differences between control and experimental groups were determined by using an unpaired *t*-test, and repeated measures multivariate analysis for intergroup and intragroup comparisons of jaw movement trajectories and EMG activities. Simple *post hoc* tests using the Sidak adjustment were performed for multiple comparisons. Statistical analysis was performed with SPSS for Windows, version 23 (SPSS, Inc., Chicago, IL, United States), and *p*-values less than 0.05 or 0.01 were considered to be statistically significant.

#### Histological Identification of Electrode Position

**FIGURE 1:**
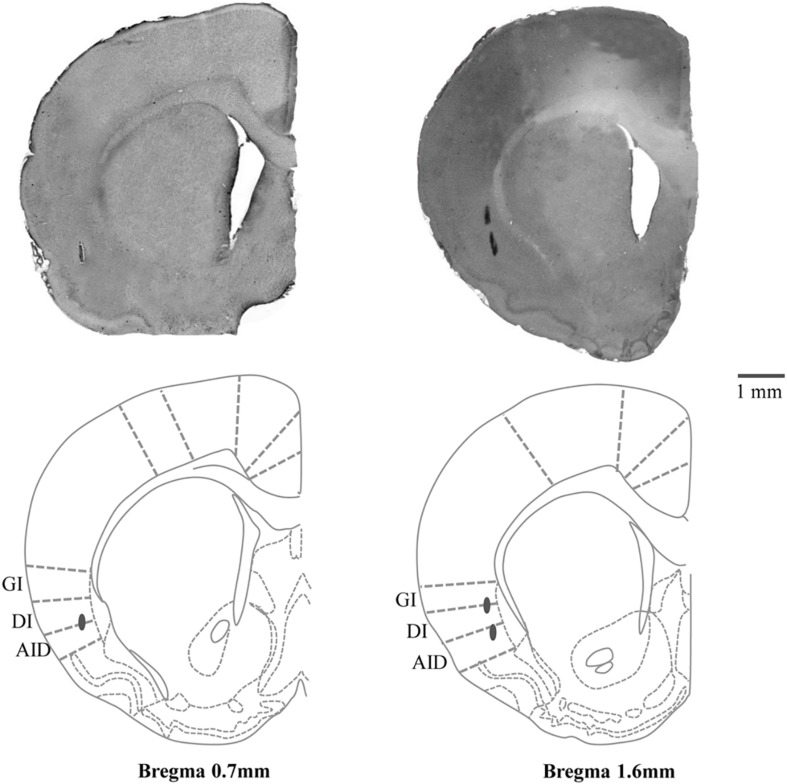
Hematoxylin-eosin stained coronal section (50 μm) with schematic drawing of the stimulation sites in the posterolateral part of left cortical masticatory area (CMA). Gray circles indicate the electrolytic lesions of the stimulation sites. Relative distances from Bregma in the rostral (0.7 and 1.6 mm anterior to the Bregma) direction are depicted. Template from the brain atlas (Paxinos and Watson, 2007), illustrating the left insular cortices (posterolateral part of CMA) showing the location of stimulation sites. GI, granular insular cortex; DI, dysgranular insular cortex; AID, dorsal part of the agranular insular cortex.

After the experiment had been completed and rats remained on the stereotaxic apparatus, they were anesthetized with Ketamine-HCl (20 mg/kg, IP) and the electrode was reinserted into some of the cortical areas at which RJMs had been induced and electrical lesions were created by passing currents (30 μA for 20 s) through the stimulating electrode. The rats were then deeply anesthetized and perfused with 100 ml of phosphate-buffered saline (PBS; pH 7.4) through the left cardiac ventricle followed by 300 ml of fixative solution of 4% paraformaldehyde. Serial coronal sections of the brain (50 μm thick) were cut and counterstained with hematoxylin-eosin stain. The locations of the electrode tips were confirmed under a light microscope and verified using the reference (Paxinos and Watson, 2007) (Figure 1).

## Results

### Body Weight

**FIGURE 2:**
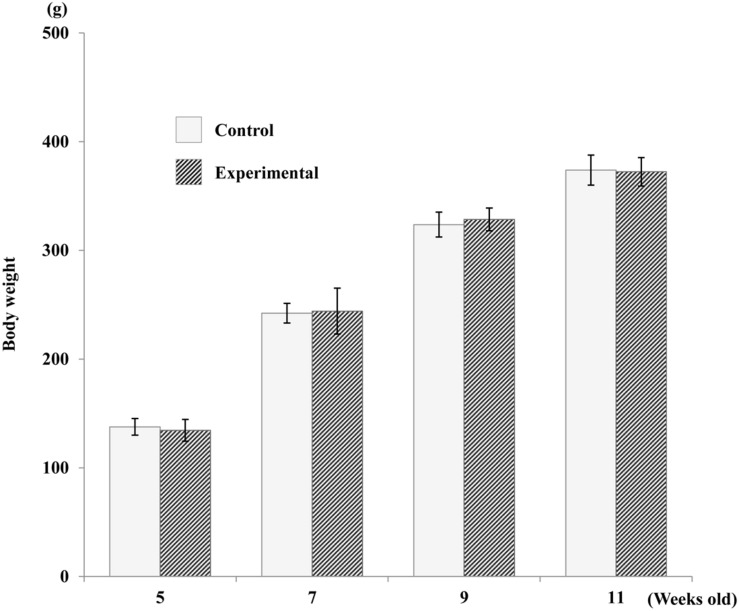
Average increase in body weight of both groups. No significant difference was found between the two groups.

Body weights of the rats in both groups increased gradually throughout the experimental period, and there were no significant differences in mean body weight between groups at any time point (Figure 2).

### Jaw Movement Trajectories During Stimulation

**FIGURE 3:**
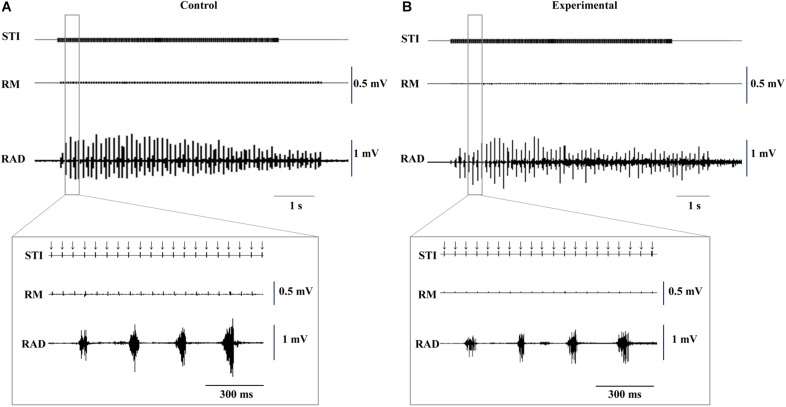
Typical example of electromyographic activity with insets of the enlarged raw data induced by electrical stimulation of the CMA at 11 weeks of age. Control **(A)** and experimental **(B)** groups. Downward arrows indicate the timing of electrical stimulation. STI, stimulation; RM, right masseter; RAD, right anterior digastric.

**FIGURE 4:**
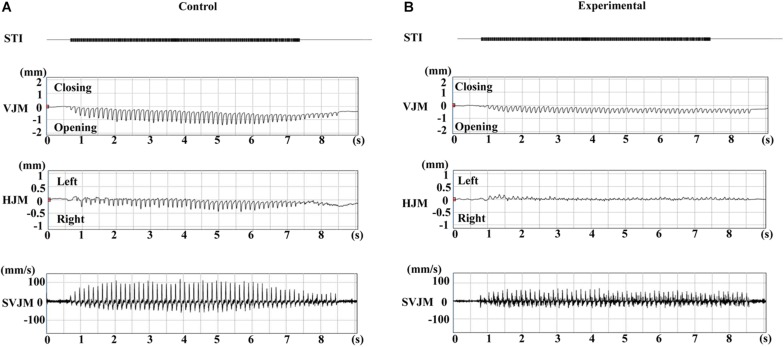
Typical jaw movement patterns induced by electrical stimulation of the CMA at 11 weeks of age. Control **(A)** and experimental **(B)** groups. STI, stimulation; VJM, vertical jaw movements; HJM, horizontal jaw movements; SVJM, speed of vertical jaw movements.

Typical EMG activity with the inset enlargement of raw data for both groups during stimulation are shown in Figure 3, and the representative recordings of RJM trajectories of both groups are shown in Figure 4. The jaw position was stable at rest and the distance between the upper and lower incisors was approximately 2–2.5 mm before stimulation.

**FIGURE 5:**
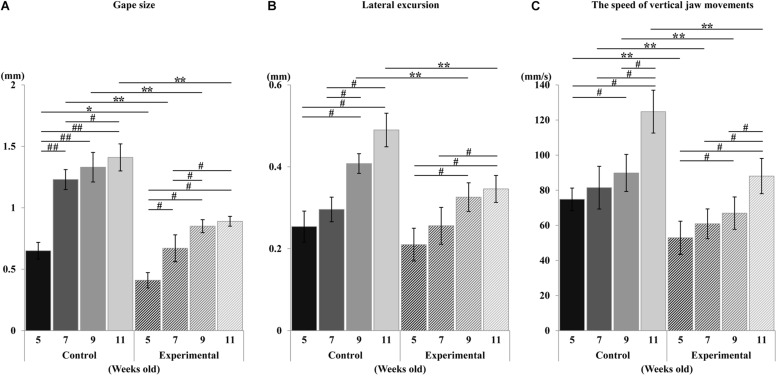
Changes in jaw movements during stimulation. Gape size **(A)**, lateral excursion **(B)**, and the speed of vertical jaw movements **(C)**. Data are displayed as mean ± standard deviation. Asterisks (*p < 0.05), (**p < 0.01) denote significant differences between control and experimental groups. Hashtags (#*p* < 0.05), (##*p* < 0.01) denote significant differences between different ages within the same group.

During the repetitive electrical stimulation, the characteristics of the jaw movement patterns and the EMG were similar to those reported in previous studies (Sasamoto et al., 1990; Satoh et al., 2007). The gape size of jaw movements in the control group was significantly larger than those in the experimental group at each recording age (Figure 5A). Relative to the control group, the gape size at weeks 7, 9, and 11 was significantly larger (*p* < 0.01) than at week 5; at week 11, it was significantly larger (*p* = 0.034) than at week 7, but there were no significant differences of gape size between weeks 7 and 9, and weeks 9 and 11. Similarly, in the experimental group, the gape size of the jaw movement at weeks 7, 9, and 11 was significantly larger (*p* < 0.05) than at week 5, and significantly larger (*p* < 0.05) at weeks 9 and 11 than at week 7, but there was no significant difference between weeks 9 and 11.

With regards to the lateral movement, the excursion in the control group was significantly larger (*p* < 0.01) than in the experimental group at weeks 9 and 11, while there was no significant difference between groups at weeks 5 and 7 (Figure 5B). Intragroup comparison of the control group showed that the lateral excursion at weeks 9 and 11 was significantly larger (*p* < 0.05) than at weeks 5 and 7, while there were no differences between weeks 5 and 7, or weeks 9 and 11. Intragroup comparison of the experimental group showed that the lateral excursion at weeks 9 and 11 was significantly larger (*p* < 0.05) than at week 5, and that of week 11 was significantly larger (*p* < 0.05) than week 7. There were no significant differences between weeks 5 and 7, or weeks 9 and 11.

The overall development of vertical mandibular speed showed an increase with age in both groups (Figure 5C). Compared to the control group, the vertical mandibular speed was significantly smaller (*p* < 0.01) in the experimental group at each recording age. Intragroup comparison within the control group showed that the speed at week 9 was significantly larger (*p* < 0.05) than at week 5, and that of week 11 was significantly larger (*p* < 0.05) than weeks 5, 7, and 9. There were no significant differences between weeks 5 and 7, or weeks 7 and 9. Similarly intragroup comparison within the experimental group showed that the speed at week 9 was significantly larger (*p* < 0.05) than at week 5, and that of week 11 was significantly larger (*p* < 0.05) than at week 5, 7, and 9. There was no significant difference between weeks 5 and 7, or weeks 7 and 9.

**FIGURE 6:**
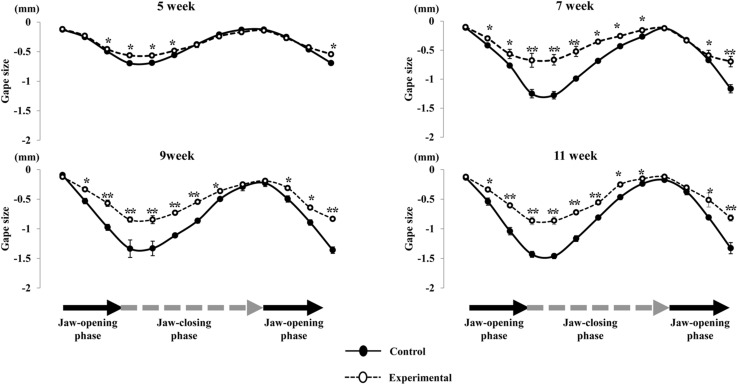
Comparison of the path of vertical jaw movement between the control and experimental groups during jaw-opening and closing phases. The 13 points mark the 10 ms intervals through the masticatory cycle. Data are displayed as mean ± standard deviation. Asterisks (*p < 0.05), (**p < 0.01) denote significant differences between the control and experimental groups.

**FIGURE 7:**
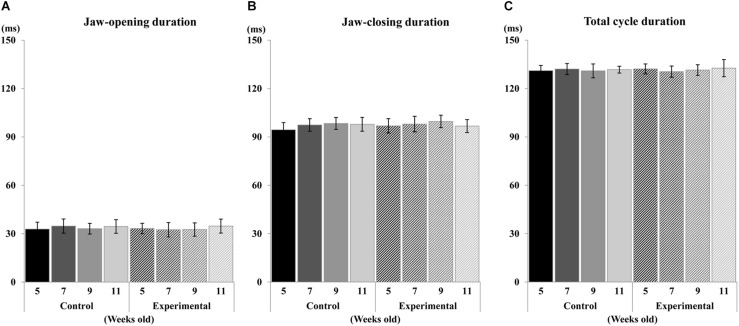
Duration of jaw movement phases during electrical stimulation. Jaw-opening duration **(A)**, Jaw-closing duration **(B)**, and total cycle duration **(C)** of control and experimental groups.

**FIGURE 8:**
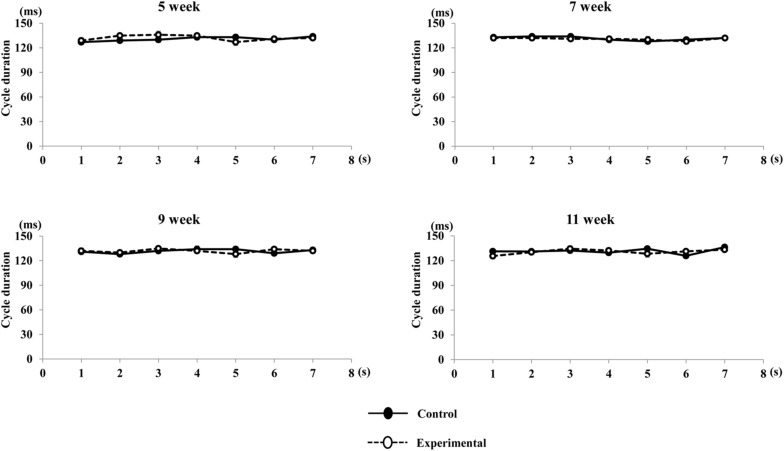
Rhythm of movement cycle duration during repetitive stimulation in each time point (per s). Rhythm of cycle durations were not significantly different in both group during repetitive stimulation every second.

Regarding the pattern of vertical jaw movement, the jaw movement paths were similar between both groups at all ages. A majority of traces overlapped along the path at week 5, except the traces at the maximum jaw-opening position, referred to as gape size, which were significantly different (*p* < 0.05) between both groups. The jaw movement paths at weeks 7, 9, and 11 were significantly increased (*p* < 0.01) in terms of gape size in the control group compared to the experimental group (Figure 6). Comparison of the jaw movement duration showed that there were no significant differences between the control and experimental groups in the jaw-opening, jaw-closing, and total cycle durations (Figure 7). Intragroup comparison revealed no significant effects of age on these parameters in either the control or the experimental group at any recording age. There were no significant differences in the rhythm of movement between the both groups at all ages (Figure 8).

**FIGURE 9:**
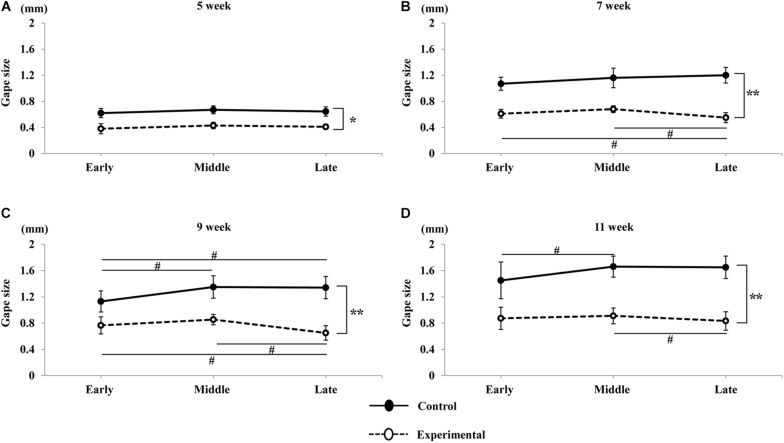
Variations in gape size of jaw movements with time in the sequence. Total duration of the stimulation sequence was divided into three equal periods: early, middle, and late, corresponding to the first, second, and third for 2 s respectively. Jaw movements at 5 weeks of age **(A)**, 7 weeks of age **(B)**, 9 weeks of age **(C)**, and 11 weeks of age **(D)** in the control and experimental groups. Data are displayed as mean ± standard deviation. Asterisks (*p < 0.05), (**p < 0.01) denote significant differences between the control and experimental groups. Hashtags (#*p* < 0.05) denote significant differences between the sequences at each age.

In terms of time-dependent changes in the jaw movement sequences, we measured and compared vertical jaw movement between the early, middle, and late periods for each age in both groups. As described above, the amplitude of vertical jaw movement, as well as the gape size, was significantly smaller in the experimental group at all ages. Comparison of vertical movements among the early, middle, and late periods at 5 weeks of age showed that there were no significant differences in either group (Figure 9A). At 7 weeks of age, there were no significant differences among the early, middle, and late periods in the control group; however, in the experimental group, the jaw movement during the late period of the sequence was significantly smaller (*p* < 0.05) than that during the early and middle periods (Figure 9B). At 9 weeks of age, the jaw movement during the middle and late period was significantly larger (*p* < 0.05) than during the early period in the control group. Conversely, the late period of the sequence in the experimental group was significantly smaller (*p* < 0.05) than the early and middle periods (Figure 9C). At 11 weeks of age, the early period of the sequence in the control group was significantly smaller (*p* < 0.05) than the middle; however, there were no significant differences between both the early and late, as well as the middle and late periods. In contrast, the late period in the experimental group was significantly smaller (*p* < 0.05) than the middle period; however, there were no significant differences between both the early and late, as well as the early and middle periods (Figure 9D).

### Electromyographic Activities During Stimulation

**FIGURE 10:**
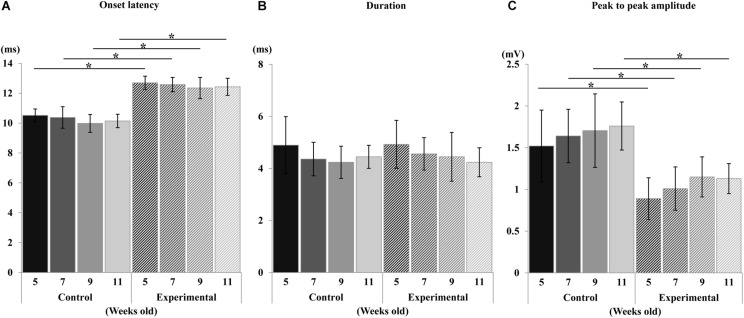
Changes in electromyographic activity. Onset latency **(A)**, duration **(B)**, and peak-to-peak amplitude **(C)** of control and experimental groups. Data are displayed as mean ± standard deviation. Asterisks (*p < 0.05) denote significant differences between the control and experimental groups.

During the sustained stimulation, the EMG activities in the anterior digastric muscle were recorded, however, no activities were seen in the masseter muscle at any age. The EMG onset latency, duration, and peak-to-peak amplitude of the anterior digastric muscles are shown in Figure 10. Compared to the control group, the onset latency was significantly longer, and the peak-to-peak amplitude was significantly smaller in the experimental group at each recording age, but there were no significant differences in intragroup comparisons at weeks 5, 7, 9, and 11 in either the control or the experimental group. In terms of duration no significant differences in EMG were observed between the two groups, or for the intragroup comparisons at any recording age.

**FIGURE 11:**
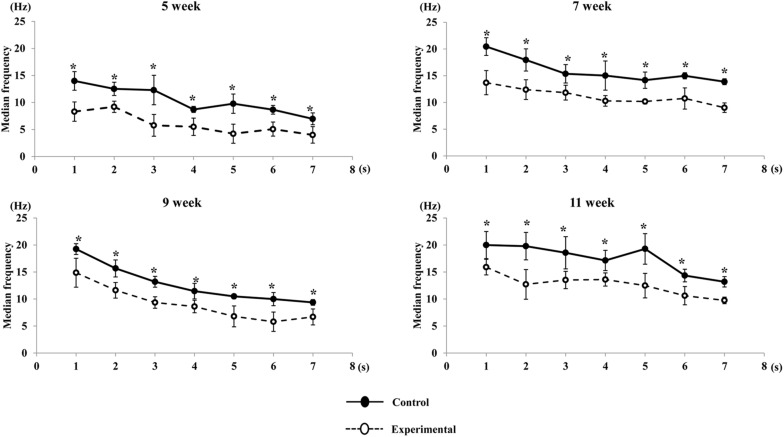
Changes in median frequency of the anterior digastric muscle during stimulation in each time point (per s) at each age. Data are displayed as mean ± standard deviation. Asterisks (*p < 0.05) denote significant differences between the control and experimental groups.

**FIGURE 12:**
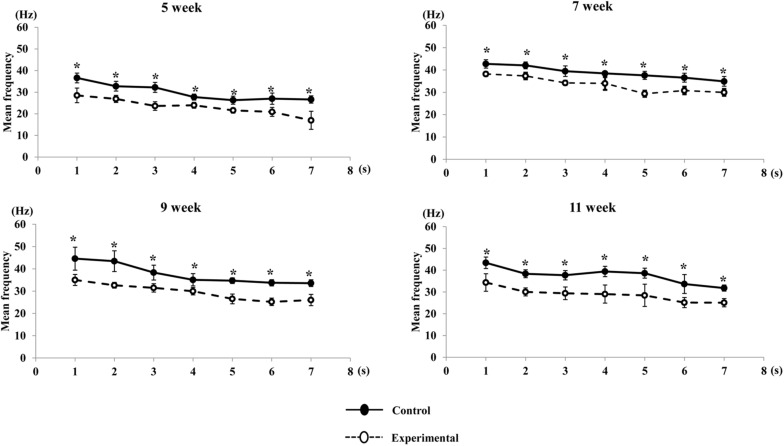
Changes in mean frequency of the anterior digastric muscle during stimulation in each time point (per s) at each age. Data are displayed as mean ± standard deviation. Asterisks (*p < 0.05) denote significant differences between the control and experimental groups.

A visual comparison of the EMG spectrum showed that a higher median frequency was observed during the early period of contraction in both groups, which shifted toward lower frequencies during the late period of contraction. The median frequency generated in the experimental group was significantly reduced compared to that of the control group at all ages (Figure 11). Similarly, the mean frequencies generated in the control group were significantly higher than those in the experimental groups during the stimulation period at all ages (Figure 12).

## Discussion

In the current study, we investigated the developmental course of cortically-evoked RJMs, comparing normal conditions to altered occlusal loading forces provided by a soft diet and assessing the neuromuscular responses of jaw-opening and jaw-closing muscles during maturation. The stimulus intensity required for CMA stimulation to elicit RJMs in rats has been shown to vary between 50 and 300 μA, and the threshold for RJMs evoked from the posterior part of the CMA is higher during repetitive stimulation (Sasamoto et al., 1990; Satoh et al., 2006b, 2007; Maeda et al., 2014; Tsujimura et al., 2016). Furthermore, it has been noted that a stimulus intensity of up to 300 μA used to evoke RJMs in rats does not appear to have any noxious effects during repetitive stimulation (Satoh et al., 2006a, b, 2007). In our study, the CMA was stimulated and RJMs subsequently identified through mandibular movements and rhythmic bursts of EMG activity. The current stimulus intensity used in our experiment was designated as the threshold required to elicit RJMs. We used a constant current strength (180 μA) to compare the cortically evoked RJMs and found that long-term alteration of the masticatory load at an early age affected the developmental course of jaw movements and the neuromuscular response of the masticatory muscle.

### Growth-Related Changes in Cortically-Induced Jaw Movements

In our study, low-frequency long-train stimulation of the posterolateral part of the CMA evoked simple vertical RJMs in both groups. The gape size of jaw movement was smallest in each group at week 5, and it increased until 9 weeks of age in both the control and the experimental groups, while there was no significant difference between weeks 9 and 11. Previous study have reported that the masticatory movements in rats appeared at approximately 3 weeks of age (Westneat and Hall, 1992). Since all repetitive neuronal bursts for rhythmical mastication were detected at approximately 13–17 days in rats (Brocard et al., 2006), the initial masticatory patterns developed at approximately 12 days, and the adult masticatory movements were established between 18 and 21 days (Westneat and Hall, 1992). However, facial morphological development continued after 3 weeks of age in rats. The variations in mandibular and condylar growth positively correlated with the maximum jaw movement capacity (Fukui et al., 2002). A recent three-dimensional study has reported that the natural growth rate of the mandible was greatest between 4 and 8 weeks of age, and reached a plateau after 9 weeks of age in rats (Kim et al., 2018). A report on the development of jaw movements in children and adolescents showed that the maximum jaw-opening movement was less stable during an early age, and increased until the age of 17 years (Hirsch et al., 2006). Thus, we considered that orofacial morphological development could affect the pattern of RJMs during maturation.

Lateral excursion movements were small at weeks 5 and 7 in the control group. During stimulation, the jaw movement deviated slightly toward the right side (i.e., the side contralateral to the stimulation) followed by grinding movements during the jaw-closing phase, but this type of movement was small at 5 and 7 weeks of age. A previous study reported that the size of the mandibular condyle and fossa did not increase between weeks 4 and 8, although body weight of the rats increased (Kato et al., 2015). Thus, the decreased lateral movements may be related to the shape of the temporomandibular joint that would restrict lateral movements at this age.

During constant stimulation, we noticed that the speed of vertical movements is augmented with age. In our study, the speed of vertical jaw movements increased until 9 weeks, then peaked at 11 weeks of age in both the control and experimental groups. A previous study suggested that the speed of the jaw movements is reflected in the amount of muscle force produced during mastication (Watanabe and Watanabe, 2001; Anderson et al., 2002). Moreover, the force and power output of a muscle mainly depends on the muscle fiber size and length. The skeletal muscle fibers of rat undergo intense growth from 3 to 10 weeks of age, with an increase in the number and size of myofibers, and attain stable growth after 10 weeks (Tamaki and Uchiyama, 1995). This suggests that changes in morphology of muscles may also affect the speed of jaw movements.

The patterns of the vertical movement were similar in both the control and experimental groups, with the exception of the gape size of jaw movements, which differed significantly with age. Many previous electrophysiological studies have reported that different sites in the CMA evoke different patterns of jaw movement (Sasamoto et al., 1990; Gerstner and Goldberg, 1991; Isogai et al., 2012; Uchino et al., 2015). Vertical RJMs have been shown to be induced by stimulating the ventrolateral area of the CMA (i.e., the agranular cortex) (Enomoto et al., 1995; Masuda et al., 2002; Isogai et al., 2012). It is likely that that stimulating the same effective site of the CMA may evoke these patterns of jaw movement.

The duration of parameters of RJMs including jaw-opening and jaw-closing showed no significant differences between ages in both the control and experimental groups. Moreover, the total cycle duration was also consistent depending on the stimulus interval during stimulation. The timing and sequence of contractions of the jaw-opening and jaw-closing muscles are associated with the development of the masticatory CPG, which generates the RJMs (Lund and Kolta, 2006; Morquette et al., 2012). It has been shown that in rats, repetitive bursting neurons are detected postnatal days 9 to 12, and rapidly increase in activity up until postnatal day 14, after which it remains constant (Brocard et al., 2006; Morquette et al., 2012). This result may suggest that the masticatory CPG maintains the cycle duration that resembles the set duration of the stimulus interval to the CMA until it reaches the mature level.

### Effects of Low Occlusal Loading on Cortically-Induced Jaw Movements

During electrical stimulation of the CMA, the gape size was significantly smaller in the experimental group than in the control group at each recording age. The difference between the gape size at week 5 in both groups had a lower level of significance than at weeks 7, 9, and 11. It has been reported that being raised with a soft diet during the optimum learning period prevents masticatory function from reaching a normal level (Fujishita et al., 2015). Our findings were consistent with this previous report, as the maximum gape size during opening did not reach a normal level in rats fed a soft diet. Moreover, the opening capacity of the jaw is associated with the coactivation of the jaw muscles and the neuromuscular response from the brain. A previous study has reported that changes in occlusal loading are responsible for the variation in the maturation of sensory afferents such as the muscle spindles and periodontal mechanoreceptors, thereby affecting the capacity for modulatory sensory feedback from the masticatory CPG (Lund and Kolta, 2006). Altered sensory inputs of the oral receptors and muscle spindles have been shown to result in decreased synaptic density in the cerebral cortex, which impairs the strengthening of synapses and causes sensory deprivation of the cerebral cortex (Yamamoto and Hirayama, 2001; Bhatt, 2010). Morphological studies have also reported that the number of cross-sectional muscle fibers and mitochondria per unit in muscles was significantly reduced in growing rats fed on a soft diet (Sato and Konishi, 2004; Kawai et al., 2010). In addition, decreases in the proportion of cross-sectional areas of both type 1 and 2 fibers in jaw-closing muscles, and type 2 fibers only in jaw-opening muscles, were found in animals during training with low occlusal force (Langenbach et al., 2003; Kitagawa et al., 2004). Changes in the oxidative capacity of muscle fibers, due to lower muscle usage after 2 weeks feeding on a soft diet, have been shown to significantly reduce the motor properties of jaw muscles in rats (Liu et al., 1998). In addition, varying masticatory loading directly affects the mandibular remodeling process, which causes changes in the morphology of the mandible (Renaud et al., 2010; Tsouknidas et al., 2017). Hence, the decreased vertical jaw movement observed in our study might be influenced by morphological changes and diminished activities of mechanoreceptors from the oro-facial regions.

The lateral excursion at 9 and 11 weeks of age was more significantly reduced in the experimental group than in the control group. Rats exhibit a smaller mandibular condyle and thinner cartilage when fed a soft diet during growth (Kato et al., 2015). Previous studies have reported that there was a regressive change in the trabecular structure when being fed a soft diet, which can reduce the resistance of the mandibular condyle to mechanical loading, and increase the occurrence temporomandibular deformation (Ogawa et al., 2016; Kono et al., 2017). In addition, alterations in occlusal stimuli followed by consumption of a soft diet may result in a decrease in the response of the mechanoreceptors of the temporomandibular joint (Ishida et al., 2009). Therefore, these findings demonstrated that the low occlusal loading during growth has a significant influence on the shape of the temporomandibular joint and its mechanoreceptors, resulting in the observed reduction in the lateral excursion.

The speed of vertical jaw movements was lower in the experimental group than in the control, at each recording age. The contraction velocity of jaw muscles is known to depend on the amount of force that is generated during muscle contraction. It has been reported that changing the consistency of food to a softer form may reduce the masticatory muscle load, and thus decrease tetanic isometric tension force during muscle contraction (Kiliaridis and Shyu, 1988). Moreover, rats raised on a soft diet show a decrease in muscle fiber conduction velocity, which delays muscle contraction time, affecting the speed of movement (Murakami et al., 2014). Human studies have also suggested that the velocity of muscle contractions during jaw-opening and jaw-closing was significantly decreased when consuming food of a softer consistency (Watanabe and Watanabe, 2001; van der Bilt and Abbink, 2017). It is possible that changing the occlusal loading with a softer diet may result in lesser masticatory force, which causes the slower speed of jaw movements observed in our study.

With regards to the similar masticatory pattern observed in our study, a human study has suggested that the path of the masticatory pattern is affected by muscle, TMJ form, and the inclination of the occlusal plane (Tomonari et al., 2017). However, rats raised on a soft diet have been shown to have a similar pattern of masticatory movement regardless of food consistency (Fujishita et al., 2015). This may be due to the different characteristics of the masticatory system between humans and rodents, since rats have limited lateral movement and diverge widely in the sagittal plane (Utsumi et al., 2010). In our study, a similar pattern of jaw movement was observed, with the exception of the gape size, which was small in rats fed a soft diet. At week 5, a majority of traces overlapped along the path between the both groups, and the low significant difference observed at the maximum gape size showed that the effect of low occlusal loading appeared 2 weeks after being fed a soft diet. The maximum gape size of jaw movement at weeks 7, 9, and 11 were more significantly reduced in rats fed a soft diet, suggesting that changes in food consistency have a larger effect on masticatory function and sensory feedback during the developmental period, resulting in a decreased gape size. However, this did not show any influence on the masticatory pattern during the electrophysiological study.

Phases of jaw movements including jaw-opening, jaw-closing, and total cycle duration were not statistically different between the experimental and the control groups. Also, no significant difference was observed in the rhythm of jaw cycle duration between both groups. When repetitive electrical stimulation is delivered to the corticobulbar tract, duration is mainly controlled by the activation of jaw-opening and jaw-closing motoneurons, which are part of the circuitry of the masticatory CPG (Morquette et al., 2012). One diet-related study reported that changing to a soft diet alters the motor unit activity of muscles due to impairment of morphological and metabolic properties of muscle, but found no changes in soma diameter and enzymatic activity of motoneurons (Miyata et al., 1993). Therefore, it appears unlikely that low occlusal loading could affect the response properties of jaw motoneurons in the masticatory CPG. Rather, stimulus of the CMA could directly activate the masticatory CPG through the corticobulbar tracts, resulting in the stable duration of jaw movements observed across both groups during stimulation in the present study.

In the present study, we divided the total duration of vertical jaw movements into 3 periods of equal duration to evaluate changes in jaw movements during constant stimulation. The amplitude of jaw movements was less stable in the experimental group during stimulation. In the late period, jaw movements during stimulation were not as marked as in the early and middle periods at 7, 9, and 11 weeks of age, which is reflected by the reduced EMG activity of the anterior digastric muscle. It has been reported that the jaw movement rhythm is mainly controlled by the activity of the jaw-opening muscles (Lund, 1991). More than 90% of fibers in the jaw-opening muscles are composed of fast-contracting or type 2 fibers (Cobos et al., 2001). In addition, a lesser proportion of type 1 and type 2B fibers, and a greater proportion of type 2A fibers have been found in developing rats fed on a soft diet (Kitagawa et al., 2004; Kawai et al., 2010). This may be due to the diminished activity of masticatory muscles resulting in a shift of fiber-type that caused conversion to type 2A fibers. Generally, type 1 fibers are optimally suited for sustained contraction force and type 2B fibers are suited for sudden and powerful contraction of large force (Grunheid et al., 2009). It is likely that the incremental shift in fiber-type may also be reflected in the stable jaw movements during stimulation, particularly in the late period.

### Growth-Related Changes and Effects of Low Occlusal Loading on Muscular Work

In our study, only activity of the anterior digastric muscle, and not the masseter muscle, was observed. Further, the anterior digastric activity during stimulation comprised of clusters that were time-locked to each stimulus pulse. Previous studies have reported that the posterior part of the CMA might correspond to the unilateral RJMs with time-locked activity of the anterior digastric muscle in guinea pigs (Gerstner and Goldberg, 1991; Enomoto et al., 1995), and that stimulation of the rostral part of the somatosensory cortex can evoke sustained jaw-opening with elevated anterior digastric activity in rats (Uchino et al., 2015). However, low-frequency long-train stimulation of the deep part of the CMA evokes RJMs with alternating activities of the anterior digastric and masseter muscles (Isogai et al., 2012). This may be associated with site dependent differences in the cytoarchitecture of the cortical areas, and the connection patterns between the granular and agranular cortex and the thalamic nuclei (Haque et al., 2010; Isogai et al., 2012); RJMs with alternating activity of the masseter and anterior digastric muscles were induced from the rostral part of granular cortex, and vertical RJMs with only the anterior digastric activity were induced from the agranular cortex (Isogai et al., 2012). It is likely that the stimulation in our study mostly induced the agranular region of the cortex, which elicited activity in anterior digastric muscle, but not the masseter muscle.

The EMG activity showed that the onset latency of the anterior digastric muscle in the experimental group was significantly delayed than in the control group, but there was no significant difference among ages in either the control or the experimental group. Measuring the onset latency is the best way to assess the muscle fiber conduction velocity during stimulation (Beck, 2006). In stimulated muscle contractions in rats, the latency was delayed at 3 weeks of age, however, the delay in latency disappeared from 5 weeks of age onwards (Seki et al., 2002; Changsiripun et al., 2012). In addition, alterations of peripheral sensory regulation due to occlusal hypofunction affect the conduction velocity of muscle fibers (Seki et al., 2002; Changsiripun et al., 2009). Therefore, we may assume that low occlusal loading caused by consumption of a soft diet may reduce the conduction velocity of the muscle resulting in delayed maturation of muscle responses, but this is not a consequence of the experimental period.

The duration of the contractions of the anterior digastric muscle did not differ between groups. During stimulated muscle contractions, the duration of jaw muscle contraction is controlled by the masticatory CPG in response to repetitive electrical stimulation of CMA (Lund, 1991; Lund and Kolta, 2006; Morquette et al., 2012). Moreover, it has been reported that altering the masticatory load does not affect the duration of muscle contraction in studies of the rat (Changsiripun et al., 2009) and humans (Anderson et al., 2002). Stimulation of the CMA induces the excitatory activation of jaw muscles through corticobulbar projections to the masticatory CPG, suggesting that the masticatory CPG maintains muscle contraction duration resulting in a stable cycle of jaw movements with differing occlusal force.

The peak-to-peak amplitude was significantly larger in the control group than the experimental group at all ages. Greater muscle force produces greater excursion of the mandible throughout the jaw movement cycle (Anderson et al., 2002). Measuring the peak-to-peak amplitude is a way to assess voluntary contraction force determined by the number of muscle fibers and the proportional number of motor units activated by electrical stimulation (Keenan et al., 2005; Kamen and Gabriel, 2010). A corresponding reduction in motor unit activity had been found with occlusal hypofunction (Miyata et al., 1993). In addition, input-output properties of the motor unit can be altered by the alteration in the functional sensitivity of oral mechanoreceptors when the masticatory conditions change (Shi et al., 2006; Ishida et al., 2009). Oral receptors, including periodontal afferents and muscle fibers, are major input sources for the CPG, exciting neurons throughout the lateral brainstem during mastication. It has been suggested that their output can be influenced by hardness-related feedback. Most of the neurons that activate during jaw-opening are excited by periodontal feedback, and the neurons that activate during jaw-closing are excited by spindle afferents (Lund and Kolta, 2006). The periodontal receptors are sensitive to changing forces, and specific changes in the distribution and shape of nerve terminals were found in periodontal afferents after changes in occlusal force (Piancino et al., 2017). A previous study has suggested that abolishing the signal information from periodontal receptors and muscle spindle receptors can completely diminish the activity of muscles and reduce the build-up speed of the masticatory force during mastication (Trulsson, 2006). In our study, the peak to peak amplitude from the anterior digastric muscle was significantly decreased in rats fed a soft diet at all ages, although we could not obtain measurements from the masseter muscle. It is believed that long-term low occlusal loading may have differential effects on burst generating neurons, resulting in decreased EMG activity during stimulation of the CMA.

In this study, we also performed a power spectral analysis with a visual representation of the power spectrum in the EMG signal. A frequency analysis of how the EMG signal varies with the time has been used to monitor muscle fatigue during muscle contraction (Cifrek et al., 2009). In this analysis, the mean and median frequencies are generally measured as a gold standard for the assessment of muscle fatigue (Phinyomark et al., 2012). The mean frequency is used to determine changes in muscle fatigue over time, whereas the median frequency measures two equal halves of the spectrum during muscle contraction (Cifrek et al., 2009). The contribution of muscle fibers to the varying size of the EMG power spectrum is associated with variations in the proportion of muscle fiber diameter. In addition, the distribution of muscle fibers during activation also affects the EMG power spectrum (Gerdle et al., 2000). It is likely that the larger the muscle fibers with fast conduction velocities, the higher the frequency in the EMG spectrogram (Seven et al., 2013). In the anterior digastric muscle of the rat, a significant proportion of type 2B, fast contracting, fibers are found in peripheral regions, and a lower proportion of type 1, slow contracting, fibers are found in the central regions (Cobos et al., 2001). The transition from type 1 to type 2 fibers may occur during reduced masticatory loading, followed by a reduction in muscle fiber size (Tsai et al., 2012). In the present study, the higher mean/median EMG burst frequencies observed during early contraction reflect the activation of larger type 2B fast contraction fibers. The shift to lower frequencies in the late contraction period suggests that a larger muscle force was generated in the early contraction period during stimulation, and the muscle force tended to reduce in the late contraction period. On the other hand, the low occlusal loading by feeding a soft diet resulted in decreased force generation during contraction, due to altered muscle fiber size and conduction velocity, which causes muscle fatigue during dynamic muscle contraction.

In summary, the findings of our study provided further evidence that decreasing occlusal loading through consumption of a soft diet induces smaller patterns of cortically-evoked jaw movements. Changes in occlusal functions are associated with neuroplasticity in the jaw and tongue motor representations within the somatosensory cortex (Avivi-Arber et al., 2010b, 2015). Neural signals from the CMA induce projections to the brainstem reticular formation and trigeminal motor nuclei, which induces various trigeminal actions to orofacial regions (Morquette et al., 2012). Cortical inputs to motoneuron pools provide a direct pathway for voluntary control of jaw muscles and fine control of voluntary bite force (Nordstrom, 2007). The masticatory CPG mainly regulates the rhythmic trigeminal activity of masticatory movements, and peripheral sensory information may modulate the central motor command (Nakamura and Katakura, 1995). Our results suggest that consuming a soft diet during early development inhibits the motor performance of higher brain centers, and masticatory functions never reach the normal levels during the experimental period. In our study, we only investigated the RJM trajectories and EMG activities from 5 to 11 weeks of age in rats. The functional properties of oral mechanoreceptors (i.e., periodontal and temporomandibular joint mechanoreceptors) and trigeminal neurons in the brainstem are mature by an age of 5 weeks in rats (Nasution et al., 2002; Ishida et al., 2009; Hiranuma et al., 2013). In addition, the craniofacial growth maturation of the rats reaches a plateau in approximately 79 days (Abed et al., 2007). Thus, we believe that it is important to learn the masticatory function and associated neuromuscular control from the brain, particularly from the CMA, around these time periods. However, it remains controversial whether the low occlusal function actually affects the functional activity of the masticatory CPG since we found no significant changes in the duration and rhythm of jaw movements between the groups in our study. Therefore, further studies are needed to explore the effects of alterations in oral functions on the functional configuration of the masticatory CPG in rats fed with foods of different consistencies.

## Conclusion

The present study shows that (1) the pattern of cortically-induced RJMs become stable after 9 weeks of age in rats; (2) changing the loading force alters the maturation and pattern of cortically induced RJMs during growth; (3) with decreased occlusal function, the masticatory CPG maintains the duration of jaw movement sequences during growth, in accordance with the stimulus interval that is applied to CMA; and (4) long-term low occlusal loading generates downward shifts in frequency with localized muscle fatigue. Collectively, the present findings suggest that alteration of occlusal loading force during the several weeks after weaning significantly modulates the regulation of cortically-induced RJMs and the neuromuscular response of jaw muscles during development. Therefore, an appropriate ingestion behavior during the growing period should be essential for the normal growth and development of the craniofacial complex, as well as for the maturation of masticatory functions.

## Data Availability Statement

The raw data supporting the conclusions of this article will be made available by the authors, without undue reservation, to any qualified researcher.

## Ethics Statement

The animal study was reviewed and approved by the Institutional Animal Care and Use Committee (A2017-135A and A2018-028A) in compliance with the Animal Care Standards of Tokyo Medical and Dental University.

## Author Contributions

All authors conceived and designed the experiments, interpreted results of experiments, approved the final version of the manuscript. PA, CK, and YA performed the experiments. PA, CK, and TOn analyzed the data. PA and CK prepared the figures and drafted the manuscript. PA, CK, SK, and TOn edited and revised the manuscript.

## Conflict of Interest

The authors declare that the research was conducted in the absence of any commercial or financial relationships that could be construed as a potential conflict of interest.
